# Accuracy of a Rapid Diagnostic Test Based on Antigen Detection for the Diagnosis of Cutaneous Leishmaniasis in Patients with Suggestive Skin Lesions in Morocco

**DOI:** 10.4269/ajtmh.18-0066

**Published:** 2018-07-09

**Authors:** Issam Bennis, Kristien Verdonck, Nora el Khalfaoui, Myriam Riyad, Hajiba Fellah, Jean-Claude Dujardin, Hamid Sahibi, Souad Bouhout, Gert Van der Auwera, Marleen Boelaert

**Affiliations:** 1 National School of Public Health, Ministry of Health, Rabat, Morocco;; 2 Department of Public Health, Institute of Tropical Medicine, Antwerp, Belgium;; 3 Department of Biomedical Sciences, Faculty of Pharmaceutical, Biomedical and Veterinary Sciences, University of Antwerp, Antwerp, Belgium;; 4 Laboratory of Parasitology, Faculty of Medicine and Pharmacy, University Hassan II of Casablanca, Casablanca, Morocco;; 5 Research Team on Immunopathology of Infectious and Systemic Diseases, Faculty of Medicine and Pharmacy, University Hassan II of Casablanca, Casablanca, Morocco;; 6 National Reference Laboratory of Leishmaniasis, Parasitology Department, National Institute of Hygiene, Rabat, Morocco;; 7 Department of Biomedical Sciences, Institute of Tropical Medicine, Antwerp, Belgium;; 8 Department of Parasitology, Hassan II Agronomy, and Veterinary Institute, Rabat, Morocco;; 9 Unit of Parasitic Diseases, Directorate of Epidemiology and Disease Control, Ministry of Health, Rabat, Morocco

## Abstract

In rural areas in Morocco, diagnosing cutaneous leishmaniasis (CL) can be challenging. We evaluated the accuracy of a rapid diagnostic test (RDT) based on antigen detection, CL Detect Rapid Test^™^ (Inbios International Inc., Seattle, WA), in this setting. We consecutively recruited patients with new skin ulcers in nine primary health centers. We took a dental broach sample for the RDT and two other tissue samples by scraping the border and center of the lesion with a scalpel and smearing it on a slide. We duplicated each smear by pressing a clean slide against it and processed the slides by microscopy, polymerase chain reaction (PCR) internal transcribed spacer 1, and kDNA minicircle PCR. In a subgroup with positive PCR, the *Leishmania* species was identified using PCR-restriction fragment length polymorphism and PCR-sequencing of *hsp70* genes. A participant with positive microscopy and/or PCR was considered a confirmed CL case. We computed sensitivity (Se) and specificity (Sp) of the RDT compared with this reference standard (ClinicalTrials.gov registration: NCT02979002). Between December 2016 and July 2017, we included 219 patients, 50% of them were under 18 years old. Rapid diagnostic test Se was 68% [95% confidence interval (CI): 61–74], Sp 94% [95% CI: 91–97], positive predictive value 95% [95% CI: 92–98], and negative predictive value 64% [95% CI: 58–70]. Despite its low Se, this novel RDT is a useful addition to clinical management of CL in Morocco, especially in isolated localities. Rapid diagnostic test–positive lesions can be treated as CL; but when RDT negative, microscopy should be done in a second step. The Se of the RDT can probably be optimized by improving the sampling procedure.

## INTRODUCTION

Cutaneous leishmaniasis (CL) is a vector-borne disease which usually leads to slowly self-healing skin lesions and residual scars on those parts of the body exposed to sandfly bites.^1^ In 2016, Morocco reported 4,951 CL cases. In the southeast of the country, CL is mainly due to *Leishmania major*, and in the central and northern regions due to *Leishmania tropica*. Some sporadic cases of CL due to *Leishmania infantum* have also been described.^2,3^ Cutaneous leishmaniasis scars on the face can cause considerable psychosocial suffering. In young women in North Africa and the Middle East, social rejection, diminished marriage and employment prospects, and suicidal thoughts have been documented.^4,5^ Women typically use traditional remedies and several cosmetics to mask the scar tissue or seek plastic surgery.^4,6^

Early diagnosis, and prompt and adequate treatment may improve the healing process and prevent the development of stigmatizing lesions and scars.^7^ However, current diagnostic methods for CL are far from optimal, and there is no single gold standard diagnostic technique. In resource-limited settings, the most used diagnostic test for CL is direct microscopy, i.e., searching for amastigote forms of the *Leishmania* parasite in Giemsa-stained skin smears or biopsies.^7^ However, the sensitivity (Se) of this test is moderate, between 53% and 76%^8–11^ depending on the disease duration, sampling technique, and skills of the microscopist.^12^ In patients with suggestive lesions and negative microscopy results, the polymerase chain reaction (PCR) can help confirm or rule out the diagnosis of leishmaniasis.^13^ In Morocco, a PCR based on the internal transcribed spacer 1 (ITS1) primer showed a Se between 84% and 100%.^14,15^ Furthermore, PCR-restriction fragment length polymorphism (RFLP) analysis of the amplification product allows species identification of the *Leishmania* parasites.^16–18^

Conditions for these diagnostic methods are not always available/optimal in the remote areas where the disease is endemic.^7^ In rural areas in Morocco, patients often have to travel up to 2 days to a center where the CL diagnosis is possible, and sometimes the laboratory request and the sample have to be sent to the national reference laboratory.^19^ Therefore, the diagnosis of CL in the *L. major*–endemic areas in the southeast is, in practice, often based on the clinical features of the lesion, whereas in *L. tropica*– and *L. infantum*–endemic areas in the northern, central, and mountainous regions, the diagnosis is more often based on a direct microscopic exam. An accurate and user-friendly rapid diagnostic test (RDT) for antigen detection would be welcome in these settings.

In general, RDTs are quick and easy to perform, with the ability to give results at the point of care, meaning that results are available during the first patient visit. Rapid diagnostic tests can be used in settings with limited laboratory infrastructure and staff.^20^ Several years after the successful development of an RDT for visceral leishmaniasis,^21–23^ an RDT for CL has been developed. The CL Detect Rapid Test^™^ (Inbios International Inc., Seattle, WA) is an antigen detection assay that detects a membrane-based amastigote antigen (peroxidoxin). The test is recommended for 10 *Leishmania* species (*L. tropica*, *Leishmania amazonensis*, *Leishmania donovani*, *L. infantum*, *Leishmania mexicana*, *Leishmania guyanensis*, *L. major*, *Leishmania braziliensis*, *Leishmania panamensis*, *Leishmania peruviana*), and is to be used on a sample taken with a dental broach from a skin ulcer not older than 4 months. Based on a study in Tunisia (A. Ben salah, unpublished data), the manufacturer claims a 100% Se for CL due to *L. major* compared with microscopy. The present study aimed to estimate the diagnostic accuracy of the CL Detect^™^ Rapid Test compared with a composite reference standard test (direct examination of skin smears by microscopy and/or PCR), in patients with skin ulcers attending primary health centers in CL endemic areas in Morocco.

## MATERIALS AND METHODS

### Study design.

This was an observational, prospective, phase III diagnostic accuracy study.

### Participants.

Study participants were recruited in nine primary health centers located in five CL endemic provinces where previous studies had established the dominant species, namely, Ouarzazate (*L. major*),^24^ Tinghir (*L. tropica*), Errachidia (*L. major*),^25^ Sefrou (*L. tropica* and *L. infantum*),^26^ and Sidi Kacem (*L. tropica* and *L. infantum*).^27^

Between December 2016 and July 2017, nurses and physicians working in these primary health centers consecutively enrolled patients with ulcerative skin lesions. To be included, patients had to have at least one ulcerative skin lesion suggestive of CL. This lesion had to be of recent onset, defined as a duration reported by the patient of less than 4 months. In case there were multiple lesions, the most recent and non-superinfected was selected for this study.

Patients were excluded if they had already received antimony treatment or any other physical (e.g., cryotherapy, thermotherapy, and laser) or traditional (e.g., burn, acid, and traditional scraping) treatment of their skin lesion during the last 2 months. Patients with apparent other diseases, or patients requiring hospitalization or any other anti-parasitic treatment were also excluded, as were those with ulcerative lesions of suspected bacterial origin which healed completely after a short course of antibiotic treatment.

### Study procedures.

After verification of the eligibility criteria and after obtaining informed consent, sociodemographic and clinical information was collected from each patient. Next, a picture was taken of the selected lesion. A health professional then took a small sample of the ulcer using a dental broach, after cleaning the skin with sterile 0.9% saline solution and discarding the crust or superficial skin with a small sterile scalpel. This first tissue sample was used for the RDT. Then, two additional skin smear samples (Mic 1 and Mic 2) were taken from the same lesion, from the center and the border of the ulcer as recommended by Suárez et al.^28^ Each smear was put on a slide and duplicated by pressing a clean slide against it. The four resulting smears were used for microscopy and PCR analyses. The slides were put in a clean and dust-free area for 1–4 hours, far from direct sunlight exposure, to make sure that they dried well at room temperature. Two slides (Mic 1 and Mic 2) were transferred to the nearest provincial laboratory for microscopy reading. The two remaining slides, resulting from the duplication, were sent to laboratories in Casablanca and Antwerp (Belgium) for PCR testing (as shown in Supplemental File).

### Index test.

**Figure 1.**
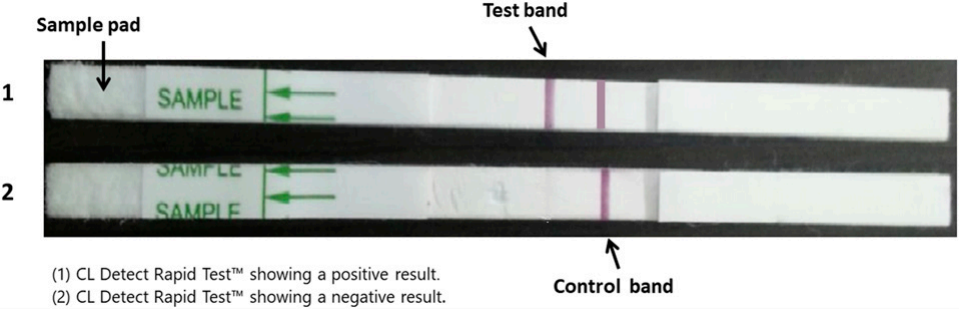
Interpretation of cutaneous leishmaniasis (CL) Detect Rapid Test^™^. This figure appears in color at www.ajtmh.org.

The CL Detect Rapid Test^™^, in package CL025 with lot identifier UA1259 and manufacturer specifications available online (http://www.inbios.com/wp-content/uploads/2016/06/900159-00-IVD-CL-Detect-Rapid-Test-Package-Insert.pdf) was done on the sample collected by the dental broach. The health professional recorded the RDT results on a separate sheet, as well as the time to perform the entire procedure from starting the RDT until reading the result, and the prevailing room temperature. We followed manufacturer’s instructions regarding reading and interpretation of results. The RDT was considered positive if both control and test bands were visible and became red, and negative if only the control band was visible (Figure 1).

### Reference standard tests.

#### Microscopy.

In the provincial laboratory, two slides per participant were fixed by dipping them in methanol for 2–3 minutes and staining them with Giemsa 10%, before examination under a light microscope (×1,000 magnification). Both slides were examined by the same microscopist, who reported the number of fields and the total number of macrophages and *Leishmania* amastigote forms found. Then, the best slide of the two (Mic1 or Mic2, depending on the ease to visualize macrophages if negative and to identify the number of *Leishmania* amastigote forms if positive) was sent to the National Institute of Hygiene (NIH) to be verified by a laboratory engineer, who had no knowledge of the patients or any previous test results. At the NIH, all slides were assessed for their quality (scored as bad, average, and excellent) according to the standard operating procedures (SOP). If the provincial laboratory reported a positive and the NIH a negative result, the other slide was also sent to the NIH. In this case, we took the conclusion from the reader at the NIH based on the two slides as the final microscopy result.

#### Internal transcribed spacer 1–polymerase chain reaction.

DNA extraction from the third slide smear was carried out manually according to the phenol–chloroform method.^14,18^ As a quality control measure, a NanoVue Plus spectrophotometer (Biochrom US, Holliston, MA) was used to measure the DNA concentration in 100 samples. As described previously,^14,18^ the ITS1 of the ribosomal DNA repeat unit was amplified in a Biometra GmbH (Whatman Biometra, Goettingen, Germany) thermocycler using primers L5.8S: 5′-TGATACCACTTATCGCACTT-3′ and LITSR: 5′-CTGGATCATTTTCCGATG-3′ (Biolegio, Nijmegen, The Netherlands). Amplification was carried out in 25 μL. Cycling conditions were as follows: denaturation at 94°C for 4 minutes, next at 94°C for 40 seconds, 53°C for 30 seconds, 72°C for 1 minute, and 72°C for 10 minutes (40 cycles). The obtained amplification products were revealed by electrophoresis on a 2% agarose gel stained with ethidium bromide and visualized under ultraviolet light. The ITS1-PCR was considered positive for the *Leishmania* genus if a band of the expected size (∼300–350 base pairs) was obtained.

For species identification, positive samples were subjected to RFLP analysis using the restriction endonuclease HaeIII (New England Biolabs, Beverly, MA).^14,18^ Reference strains of *L. infantum* (MHOM/ES/90/LEM2205), *L. tropica* (MHOM/MA/2010/L02), and *L. major* (MHOM/IL/81/Friedlin) were included.

#### kDNA-polymerase chain reaction.

Two slides per lesion were used for molecular testing. One of the two slides was systematically analyzed by using ITS1-PCR in Casablanca and the second slides were divided into two groups to be analyzed by ITS1-PCR in Casablanca or by kDNA-PCR in Antwerp as explained in supplemental file 1. The kDNA-PCR was used as an additional method to detect *Leishmania* DNA in the samples. Because this PCR amplifies the parasite’s kinetoplast DNA minicircles, of which 10,000–20,000 copies are found in each cell, it is more sensitive than ITS1-PCR. DNA was extracted using either the phenol–chloroform method described previously or (for most samples) using the QiaAmp DNA Mini Kit (Qiagen, www.qiagen.com). Lysis was achieved by 3 hours incubation at 56°C in tissue lysis buffer, and the DNA was eluted in 50 μL elution buffer AE. Primers JW11 and JW12 were used^29^ in a reaction of 25 μL containing 1x QuantiTect SYBR Green PCR Master Mix (Qiagen); 1 μM of each primer; 0.1 mg/mL acetylated BSA; and 2.5 μL DNA. Cycling was performed in a Light Cycler 480 instrument (Roche, molecular.roche.com) using an initial denaturation of 15 minutes at 95°C, followed by 50 cycles of 10 seconds at 95°C, 20 seconds at 57°C, and 30 seconds at 72°C. The Tm of the amplicons was determined in a melting curve analysis from 67°C to 95°C. A standard curve was included in each PCR, ranging from 10^3^ down to 10^−4^ pg *L. infantum* DNA, determined spectrophotometrically. The kDNA-PCR was considered positive for *Leishmania* if more than 0.2 parasite genome equivalents were detected on the slide, and a melting peak was observed between 79.5°C and 81°C.

### Analysis.

Data were entered in duplicate in Microsoft Excel 2016 and a duplicate check was performed to detect typing errors. Data analysis was performed using Stata 13 (Stata Corporation, College Station, TX). Differences in proportions were tested using Fischer’s exact test. The Se, specificity (Sp), positive predictive value (PPV), and negative predictive value (NPV) of the RDT were calculated comparing the results of the index test to the composite reference standard. The composite reference standard was defined as follows: A patient was considered as a confirmed case of CL if microscopy results and/or PCR results (be it kDNA or ITS1) were positive. Otherwise, the patient was considered a non-case. Test accuracy was first calculated in the whole study population and then compared across subgroups defined on the basis of geographical origin, *Leishmania* species, and slide quality.

We estimated the required sample size (*N* = 240) based on an expected frequency of CL in the patient series of 50%, an anticipated Se of 97% and Sp of 84% of the CL Detect Rapid Test^™^, an α error of 5%, and a power of 80%.

### Quality assurance.

The study was registered on ClinicalTrials.gov with the identifier NCT2979002 in November 2016. Before the start of the study, SOPs were developed (available on request). These SOPs were carefully explained and validated during three training days for all health professionals involved in the study procedures. The diagnostic tests in this study were done blinded to the results of other tests, i.e., readers of index and reference tests did not have access to any other test results. Each package of RDT was subject to internal quality control, using a positive and negative control following the manufacturer’s recommendations. These controls of all RDT packages were performed during a training workshop before the study. We report the results in compliance with the STARD (Standards for Reporting of Diagnostic Accuracy studies) checklist.^30^

### Ethical aspects.

The protocol of this study was approved by the Institutional Review Board of the Institute of Tropical Medicine in Antwerp (Reference1060/15), the Ethics committee of Antwerp University Hospital UZA (Reference15/51/557), and the Comité d’éthique en recherche biomedicale CERB (Reference41/16) in Rabat. The results of the index test under evaluation and the molecular tests (PCR) were not communicated to the patients or their physicians. Hence, no therapeutic decisions were based on the index test results, and CL case management followed current clinical guidelines in Morocco.

## RESULTS

### Participants.

**Figure 2.**
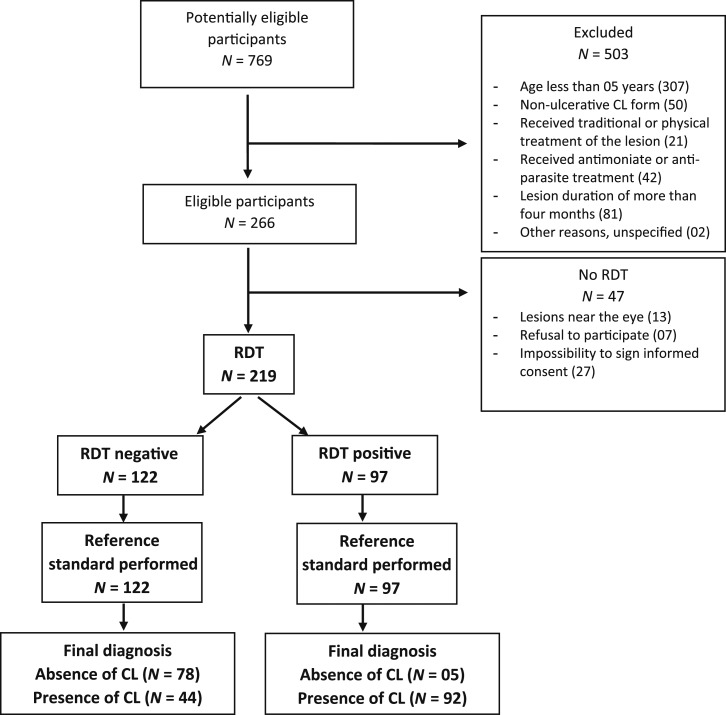
Flowchart of the study.

In total, 219 patients were included (Figure 2), among whom there were 113 men (52%) and 106 women (48%). Half of the participants (*N* = 108; 50%) were under 18 years old. Seventy-four patients (34%) had 2–10 lesions. The diameter of the selected lesions ranged between 0.1 and 5 cm. Most lesions were small: 142 participants (65%) had lesions with a diameter of 1 cm or less. Table 1 shows the demographic and clinical characteristics of the study population. None of the health professionals reported any adverse events from the sampling, even if they did not systematically use lidocaine ointment (Emla^®^ AstraZeneca, Ukkel, Belgium) or injectable xylocaine. There were no withdrawals from the study.

**Table 1:** Demographic and clinical characteristics of 219 patients enrolled

Characteristics		*N*
Provinces historically endemic for *Leishmania major*		
Errachidia	–	42
Ouarzazate	–	35
Provinces historically endemic for *Leishmania tropica*		
Tinghir	–	65
Sefrou	–	67
Sidi Kacem	–	10
All	–	219
Patient age (years)*	[5–17]	108
	[18–87]	110
Gender	Women	106
	Men	113
Lesion on the face	Yes	112
	No	107
Lesion duration (days)†	≤ 50	105
	> 50	107
Lesion diameter (cm)*	≤ 1	142
	> 1	76
Number of lesions per patient	1	145
	2–10	74

* Information missing for one participant.

† Information missing for seven participants.

### Test methods.

For the RDT, there were no missing data, nor any invalid results. The RDT gave 97 positive and 122 negative results. The average time to perform the RDT was 32 ± 7 minutes (*N* = 217) with a range between 15 and 48 minutes.

For the reference standard tests, some data were (partially) missing, as during transportation of microscopy slides between the primary health facilities and provincial laboratories, 17 slides broke and one of them was irrecoverable. Moreover, 32 slides were not well fixed, which limited the second reading by the NIH supervisor. For PCR tests, three partially broken slides were recoverable and two completely irrecoverable. The mean time (±SD) to obtain the result of microscopy from the provincial laboratories was 2.7 ± 3 days (*N* = 198).

### Diagnostic accuracy.

**Figure 3.**
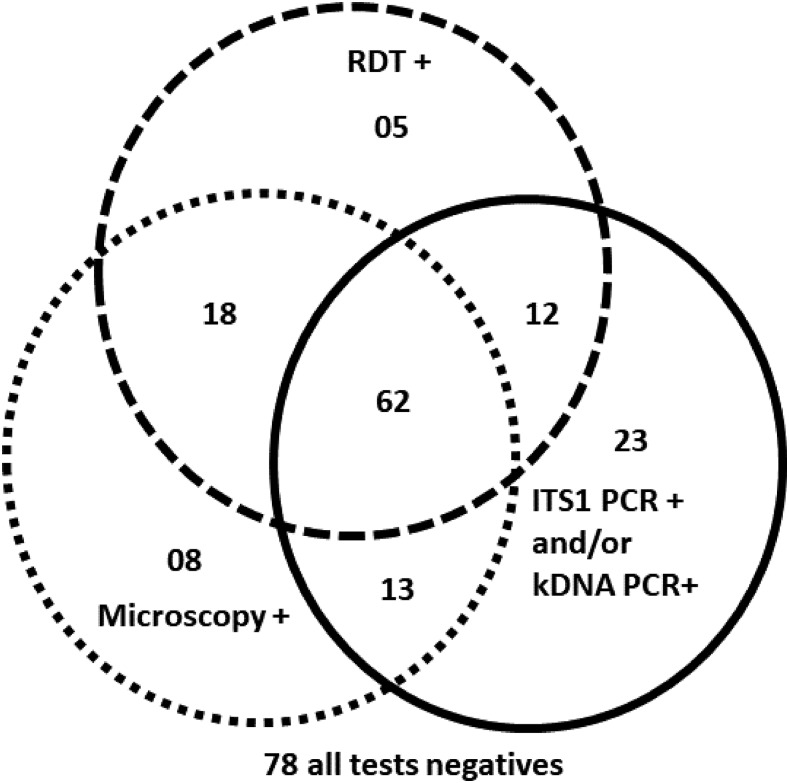
Distribution of index and reference test results in the study population (*N* = 219).

The reference standard classified 136 (62%) participants as CL cases: 75 had both positive microscopy and positive PCR results, 26 had positive microscopy only, and 35 had positive PCR only (Figure 3). The frequency of confirmed CL in this patient series varied by region: in the areas known to be endemic for *L. major*, it was 70%, compared with 58% in *L. tropica*–endemic regions. The *Leishmania* species could be identified in samples of 87 participants: 35 were *L. major*, 40 *L. tropica*, and 10 *L. infantum*. The geographical distribution of the species is shown in Table 2.

**Table 2:** Place of inclusion and index test results according to *Leishmania* species, *N* = 87

	*Leishmania major*		*Leishmania infantum*		*Leishmania tropica*	
	*N*	RDT +	*N*	RDT +	*N*	RDT +
Provinces historically endemic for *L. major*						
Errachidia	19	12	0	–	0	–
Ouarzazate	14	10	0	–	0	–
Provinces historically endemic for *L. tropica*						
Tinghir	2	1	1	1	22	16
Sefrou	0	–	10	8	18	17
Sidi Kacem	0	–	1	1	0	–
Total	35	23	12	10	40	33

RDT = rapid diagnostic test. Species identification based on internal transcribed spacer 1 restriction fragment length polymorphism (*N* = 80) or *HSP70* (*N* = 7).

When compared with the composite reference standard, the RDT showed a Se = 68% [95% confidence interval (CI): 61–74], Sp = 94% [95% CI: 91–97], PPV = 95% [95% CI: 92–98], and NPV = 64% [95% CI: 58–70] in the whole study population (*N* = 219). The diagnostic accuracy varied according to the geographical origin of the samples, although this difference was not statistically significant. The Se of the RDT was higher in regions historically endemic for *L. tropica*, i.e., 73% [95% CI: 66–80] versus 59% [95% CI: 48–70] in *L. major*–endemic regions (Table 3). This species effect was supported by comparing the results of the RDT after typing of the samples: 83% of the confirmed *L. tropica* and 66% of the *L. major* samples were RDT positive (Table 2); However, in this comparison, the observed differences in Se and Sp did not reach statistical significance (Fisher exact = 0.32 and 0.10, respectively). On the other hand, we did find a significant association between RDT accuracy and smear slide quality: RDT Se was significantly higher among patients in whom microscopy slides of better quality were obtained compared with the rest of the population (Table 3) (Fisher exact = 0.001).

**Table 3:** Accuracy of the CL Detect RDT^™^ according to geographical origin of the samples and slide quality


	*N*	Sensitivity	Specificity	Positive predictive value	Negative predictive value
Provinces historically endemic for					
*Leishmania tropica*	142	73% [95% CI: 66–80]	92% [95% CI: 87–96]	92% [95% CI: 88–97]	71% [95% CI: 64–79]
*Leishmania major*	77	59% [95% CI: 48–70]	100%	100%	51% [95% CI: 40–62]
Slide quality*					
Excellent quality	42	88% [95% CI: 78–98]	100%	100%	71% [95% CI: 58–85]
Average quality	92	64% [95% CI: 54–74]	96% [95% CI: 92–100]	98% [95% CI: 95–100]	50% [95% CI: 40–60]
Bad quality	69	53% [95% CI: 42–65]	90% [95% CI: 83–97]	80% [95% CI: 71–89]	72% [95% CI: 61–82]

CI = confidence interval; CL = cutaneous leishmaniasis; RDT = rapid diagnostic test.

* Sixteen cases were impossible to assess by the laboratory engineer at the National Institute of Hygiene because of the poor fixation of the smear slides.

The room temperature during the RDT implementation varied between 8°C and 38°C. Nine RDTs were performed at a room temperature above 30°C. In total, only 47 samples (21%) were examined at a room temperature between 20° and 30°C, that is, corresponding to the manufacturer’s instructions.

## DISCUSSION

We report the first evaluation of an RDT for CL in a series of Moroccan patients with skin lesions suggestive of CL. In remote and rural endemic areas in Morocco, we can recommend for patients presenting skin ulcers suggestive of CL to use, at first, the RDT, followed by microscopy for those who are RDT negative. Indeed, the Se of this RDT was moderate, and the Sp was excellent, leading to a high PPV of 95%. This means that 19 of 20 patients with an RDT-positive ulcer truly have CL and that treatment is, therefore, warranted in this group. The RDT-negative patients will need a second test to rule out CL, as the NPV was low. The turn-around time (i.e., the rapidity of getting the results) of less than 40 minutes, the absence of invalid results, and the short time required for training the health center staff are also supporting the use of this RDT in remote rural areas. The study followed a phase III design, recruiting suspect patients in real-life conditions in nine different health facilities, and this is representative of the context in which the RDT will be used in the future. This study design provides a solid basis for eventually reformulating the diagnostic policy in CL in Morocco.

Current limitations of this RDT are that its stability in ambient temperature is only assured for 2 years, as well as the cost. In Morocco, the current (2016) unit price of this RDT was US$ 8 per patient, which is not affordable in comparison with microscopy that costs less than US$ 1 per patient. However, a recent study in Sri Lanka using the same RDT for *L. donovani* mentioned a lower cost, US$ 4 per patient,^31^ and future cost may evolve as a function of demand and offer.

We acknowledge that our study has some limitations. First, our reference standard classification was probably not 100% perfect. The sampling procedures for microscopy and PCR were the main factor that may have limited the Se of the reference standard. Second, the kDNA-PCR was not systematically performed on all the second slides. A suboptimal Se of the reference standard may have decreased somewhat the Sp estimate of the RDT, although this seems not so apparent from our results. Third, it was not possible in this study to systematically determine the alternative diagnoses in patients who did not have CL. Fourth, because the age of lesions was based on the subjective declaration of the patient, we may have unwittingly included a few patients with lesions older than 4 months.^12^

Our results are consistent with those from a study in Afghanistan, in which the same CL RDT was used in 257 patients with mostly nodular lesions, living in Kabul, where *L. tropica* is known to be the predominant species.^32^ The accuracy of the RDT compared with a reference standard based on qPCR and microscopy was: Se = 65% [95% CI: 59–71]; Sp = 100%; PPV = 100% [95% CI: 98–100]; and NPV = 16% [95% CI: 9–24].^32^

An economic evaluation of the same RDT compared with microscopy in a population living in *L. tropica* areas, reported that the incremental cost-effectiveness ratio per DALY averted was less than US$ 10.15 for the RDT compared with microscopy, which makes it an attractive option.^33^ Other studies suggest that loop-mediated isothermal amplification (LAMP) could be an alternative as a point-of-care diagnostic, although the required equipment, training, and technology is less user-friendly than an RDT. Reverse transcriptase LAMP seems especially interesting for CL diagnosis in regions where a species-specific diagnosis is required because several species circulate simultaneously.^34–37^

Some questions remain unanswered. The study was not powered to show any species-dependent differences in Se, although our results suggest there is such an effect. If this is explored further and a species effect is confirmed, further work would need to verify if the antigen targeted by the assay is not slightly different in *L. major*–endemic areas in Morocco. In the Sri Lanka study, the low Se of the RDT (36%) was linked to the low expression of the peroxidoxin antigen in Sri Lankan *L. donovani*.^31^ Alternatively, the species effect could simply be due to the differences in parasite load, which could obviously influence the assay’s performance. The moderate Se of the RDT in this study could also be explained by a low parasite yield within the sample taken by the small saws of a dental broach. There is scope for optimizing the sampling technique, for example with the use of a sterile disposable punch, as recently published, or by repeating the procedure.^38^ Further work is required to explore these hypotheses.

In conclusion, this novel RDT for CL in its current format is a useful addition to clinical case management in Morocco, especially in isolated localities far from provincial laboratories. A positive RDT has a very good PPV (95%) and warrants treatment in CL endemic areas. This RDT is a powerful decision-making tool for nurses and other health professionals to improve the management of CL in remote areas. The RDT can be part of an algorithm that uses microscopy in a second step. Nevertheless, we recommend improving the Se of this RDT, and this could probably be reached by optimizing the sampling procedure.

## Supplementary Material

Supplemental file
