# Supplementary material for: Accuracy of a Rapid Diagnostic Test Based on Antigen Detection for the Diagnosis of Cutaneous Leishmaniasis in Patients with Suggestive Skin Lesions in Morocco
Source: Am J Trop Med Hyg. 2018 Jul 9;99(3):716–22. doi: 10.4269/ajtmh.18-0066 (PMC6169188; doi:10.4269/ajtmh.18-0066)
Supplement: Supplementary file 1 [file tpmd180066.SD1.doc]

**Mic 1 =** S1

first sample by scraping

S3 duplicate of S1

**Mic 2 =** S2

second sample by scraping

S4 duplicate of S2

**Dental broach**

**sample for RDT**

SECOND PCR on S3: half ITS1 (Casablanca) and half kDNA (Antwerp)

FIRST PCR on S4: (ITS1) (Casablanca)

Microscopy reading of S1 and S2

**Or**

SECOND PCR on S4: half ITS1 (Casablanca) and half kDNA (Antwerp)

FIRST PCR on S3: (ITS1) (Casablanca)

Microscopy reading of S1 and S2
